# Global Efficiency of Structural Networks Mediates Cognitive Control in Mild Cognitive Impairment

**DOI:** 10.3389/fnagi.2016.00292

**Published:** 2016-12-15

**Authors:** Rok Berlot, Claudia Metzler-Baddeley, M. Arfan Ikram, Derek K. Jones, Michael J. O’Sullivan

**Affiliations:** ^1^Division of Neuroscience, Institute of Psychiatry, Psychology and Neuroscience, King’s College LondonLondon, UK; ^2^Department of Neurology, University Medical Centre LjubljanaLjubljana, Slovenia; ^3^Cardiff University Brain Research Imaging Centre (CUBRIC), School of Psychology, and the Neuroscience and Mental Health Research Institute, Cardiff UniversityCardiff, UK; ^4^Departments of Epidemiology, Radiology, Neurology, Erasmus MC, University Medical Center RotterdamRotterdam, Netherlands; ^5^Mater Centre for Neuroscience and Queensland Brain Institute, University of QueenslandBrisbane, QLD, Australia

**Keywords:** cognitive aging, cognitive control, mild cognitive impairment, tractography, neuroimaging, diffusion MRI, networks

## Abstract

**Background:** Cognitive control has been linked to both the microstructure of individual tracts and the structure of whole-brain networks, but their relative contributions in health and disease remain unclear.

**Objective:** To determine the contribution of both localized white matter tract damage and disruption of global network architecture to cognitive control, in older age and Mild Cognitive Impairment (MCI).

**Materials and Methods:** Twenty-five patients with MCI and 20 age, sex, and intelligence-matched healthy volunteers were investigated with 3 Tesla structural magnetic resonance imaging (MRI). Cognitive control and episodic memory were evaluated with established tests. Structural network graphs were constructed from diffusion MRI-based whole-brain tractography. Their global measures were calculated using graph theory. Regression models utilized both global network metrics and microstructure of specific connections, known to be critical for each domain, to predict cognitive scores.

**Results:** Global efficiency and the mean clustering coefficient of networks were reduced in MCI. Cognitive control was associated with global network topology. Episodic memory, in contrast, correlated with individual temporal tracts only. Relationships between cognitive control and network topology were attenuated by addition of single tract measures to regression models, consistent with a partial mediation effect. The mediation effect was stronger in MCI than healthy volunteers, explaining 23-36% of the effect of cingulum microstructure on cognitive control performance. Network clustering was a significant mediator in the relationship between tract microstructure and cognitive control in both groups.

**Conclusion:** The status of critical connections and large-scale network topology are both important for maintenance of cognitive control in MCI. Mediation via large-scale networks is more important in patients with MCI than healthy volunteers. This effect is domain-specific, and true for cognitive control but not for episodic memory. Interventions to improve cognitive control will need to address both dysfunction of local circuitry and global network architecture to be maximally effective.

## Introduction

Cognitive or executive control describes the marshaling of cognitive resources in the face of complex or competing demands (Shenhav et al., 2013). Impairment of control is an important feature of dementia (Royall et al., 1998) and is associated with changes in brain structure. We have previously shown that alterations in a single portion of the anterior cingulum bundle predict variation of cognitive control in healthy older people (Metzler-Baddeley et al., 2012a). This observation fits with a key role for the dorsal anterior cingulate cortex (Shenhav et al., 2013). However, this is only one node of a widely distributed network that is activated by control tasks (Cole and Schneider, 2007). Alterations in brain structure occur at multiple levels with aging and early neurodegeneration. An alternative viewpoint, therefore, is that performance might depend on emergent properties of the whole network rather than any single tract. The relationship between alterations at the level of tracts and whole networks, and their relative contribution to cognitive performance in aging and neurologic disease, are not known.

Cognitive control and episodic memory have traditionally been associated with structures in the prefrontal cortex and medial temporal lobe, respectively (Alexander et al., 2007; Gläscher et al., 2012). This anatomical parcellation of function extends to key white matter connections. Cognitive control is exquisitely sensitive to microstructural differences in subsets of pathways within the cingulum bundle, including those likely to terminate in the dorsal anterior cingulate cortex (Metzler-Baddeley et al., 2012a). It is not, however, associated with variations in fornix microstructure, the principal correlate of verbal recall (Metzler-Baddeley et al., 2011). In Mild Cognitive Impairment (MCI), the prodromal stage of Alzheimer’s disease, microstructure is compromised in the fornix and other temporal tracts and residual memory performance remains dependent on temporal lobe connections (Metzler-Baddeley et al., 2012b). Performance, therefore, has been linked with relative specificity to microstructure of white matter connections within relevant networks.

Graph theory provides a means to derive properties of the brain’s global ‘connectome’, such as measures of efficiency of network structure and clustering of network nodes (Rubinov and Sporns, 2010). *Global efficiency* is inversely related to topological distance between nodes and is typically interpreted as a measure of the capacity for parallel information transfer and integrated processing (Bullmore and Sporns, 2012). The *clustering coefficient* is a measure more weighted to the local environment of each node, as it quantifies the extent to which neighboring nodes are connected to each other (Bullmore and Sporns, 2009). Reduced efficiency of network structure has been demonstrated in Alzheimer’s disease and linked to performance in both memory and executive tasks (Lo et al., 2010; Reijmer et al., 2013). In MCI, similar alterations in structural network topology have been observed, though findings at this early stage of neurodegeneration are less consistent (Bai et al., 2012; Shu et al., 2012).

Previous neuroimaging studies have generally not considered both ‘local’ (nodes and connections) and ‘global’ (network topology) measures together. To date, diffusion MRI studies have tended to focus either on detailed tract reconstructions or whole-brain approaches. It remains unclear how microstructural changes in single tracts relate to global network topology, and how important such a pathway of effect might be in cognitive function and dysfunction. This is a particularly relevant question for cognitive control. The cingulate cortex and its connections harbor critical functional specialization, but the cingulum also provides a pathway of communication across large-scale networks whose topology might also relate to cognition.

The interplay between local tracts and global network properties – and the spatial scale of organization that is most relevant to performance – have important implications for treatment. Treatments based on noninvasive stimulation could target specific local alterations in function, or the restoration of more widespread patterns of network structure and function. For example, transcranial magnetic stimulation has been shown to normalize functional connectivity in depression (Liston et al., 2014), and transcranial direct current stimulation also influences resting-state networks (Peña-Gómez et al., 2012). This study combined investigation of critical tracts with global properties of structural networks. We determined whether network topology was altered in MCI and whether such alterations were an independent factor in cognitive performance. Mediation analyses were used to test the hypothesis that relationships between tract microstructure and cognition were mediated by alterations in global network topology.

## Materials and Methods

### Participants

Twenty-five patients with MCI were recruited from the Cardiff Memory Clinic. Standardized assessment included clinical history, ascertainment of vascular risk status, neurological examination, basic hematology and biochemistry investigations, neuroimaging with CT or MRI and cognitive screening with the Addenbrooke’s Cognitive Examination (Mioshi et al., 2006). Diagnosis of MCI was based on established current criteria (Albert et al., 2011). Objective memory impairment was confirmed by a score of >1.5 SDs below age-matched controls on either the Addenbrooke’s verbal memory subscore or the visual memory test from the Repeatable Battery for the Assessment of Neurological Status. All patients had a Mini-Mental State Examination score of ≥24 (mean 26, SD 1.7) and a Clinical Dementia Rating of 0.5. Seven patients had additional evidence of executive dysfunction (multidomain MCI), others had pure amnestic MCI. Consecutive patients, who were eligible and willing to take part, were recruited and assessed by a single neurologist (MJO).

The 20 healthy control participants were drawn from 46 individuals between the ages of 53 and 93 years, recruited for an aging study (Metzler-Baddeley et al., 2011). Among the 46 elderly participants, one withdrew and another did not complete the study due to ill health. One participant was excluded because of subsequent diagnosis of Parkinson’s disease. Structural MRI scans (fluid-attenuated inversion recovery and T1-weighted) were inspected for overt pathology: three participants were excluded because of extensive white matter hyperintensities suggestive of significant cerebral small vessel disease (Fazekas grade 3) (Fazekas et al., 1993), and one participant was excluded due to severe motion artifact. From remaining 39 subjects, a matched control group was sampled. The control sample were matched for age and premorbid IQ using data from the National Adult Reading Test-Revised (NART-R), an accepted measure of premorbid IQ. Age and NART-R only were used to select this group and to prevent bias, selection was performed blind to cognitive, clinical and MRI data. Participants older than 65 years (the MCI group were all over 65) and with a verbal IQ not exceeding 2 SDs above the average patient IQ in the NART-R provided a matched sample of 20 healthy control participants.

Exclusion criteria for both groups were: a history of neurological disease or mental disorders (clinical disorders or acute medical conditions/physical disorders, as defined by DSM-IV-TR), including past history of moderate to severe head injury, prior or current drug or alcohol abuse, previous large-artery stroke or cerebral hemorrhage, known cervical, peripheral or coronary artery disease, structural heart disease or heart failure, and contraindications to MRI. Anxiety or antidepressant use was not an exclusion criterion, unless an individual met criteria for major depression. No patient with MCI met diagnostic criteria or had characteristic clinical features to suggest other degenerative disorders. An additional exclusion criterion for healthy participants was the past or current presence of subjective memory symptoms.

Ethical approval for the study was provided by the South East Wales Research Ethics Committee. All participants provided informed consent in accordance with the Declaration of Helsinki.

### Cognitive Assessment

Neuropsychological assessment was performed over two 1.5-h testing sessions. Cognitive control was assessed with tasks that required the maintenance of a task set under speeded response conditions: attention switching was examined using alternation between letters and digits with a Verbal Trails Test. The Stroop Color-Word test was used to assess the suppression of response incongruent information (Trenerry et al., 1989). Verbal generation and fluency were measured with the verbal fluency tests from the D-KEFS for letters F, A, and S and for the categories of animals and boys’ names. Motor planning skills based on spatial rules were assessed with the Tower of London test from the Delis and Kaplan Executive Function System battery (D-KEFS). The Digit Symbol Substitution test from the WAIS-III provided a measure of focused attention and psychomotor performance.

Free recall was assessed with the Free and Cued Selective Reminding Test (Grober et al., 1997). Additionally, the face recognition test from the Camden Recognition Memory Test (CRMT) was performed.

### MRI Acquisition

Diffusion-weighted MRI data were acquired using a 3T GE HDx MRI system (General Electric) with a twice-refocused spin-echo echo planar imaging sequence, providing whole oblique axial (parallel to the commissural plane) brain coverage (60 slices, 2.4 mm thickness, field of view 23 cm, acquisition matrix 96 × 96). Acquisition was peripherally gated to the cardiac cycle. TE (echo delay time) was 87 ms and parallel imaging (array spatial sensitivity encoding (ASSET) factor 2) was used. The *b*-value was 1,200 s/mm^2^. Data were acquired with diffusion encoded along 30 isotropically distributed directions and 3 non-diffusion-weighted scans, according to an optimized gradient vector scheme (Jones et al., 1999). Acquisition time was approximately 13 min.

T_1_-weighted structural MRI data were acquired using a 3D fast spoiled gradient recalled (FSPGR) echo sequence (matrix of 256 × 256 × 176, field of view of 256 mm × 256 mm × 176 mm, resulting in isotropic 1 mm resolution). The timing parameters were TR/TE/TI = 7.9/3.0/450 ms, and the flip angle was 20°.

### Image Processing and Tractography

The acquired diffusion-weighted images were corrected for distortion and motion artifacts with reorientation of encoding vectors (Leemans and Jones, 2009) and modulation of the signal intensity by the Jacobian determinant of the transformation (Jones and Cercignani, 2010). The free-water elimination approach was used to correct for atrophy-related partial volume effects due to CSF contamination (Pasternak et al., 2009; Berlot et al., 2014).

Whole-brain tractography was performed using ExploreDTI^1^ and a diffusion tensor model using every voxel as a seed point. A deterministic tracking algorithm estimated the principal diffusion orientation at each seed point and propagated in 0.5 mm steps along this direction. The fiber orientation was then estimated at the new location and tracking moved a further 0.5 mm along the direction that subtended the minimum change of principal direction. A streamline was traced until fractional anisotropy fell below 0.15 or the change in direction exceeded 60°.

Three-dimensional reconstructions of the cingulum and of temporal association tracts were derived. Detailed reconstruction algorithms and linked reproducibility data, showing good reproducibility, have been described previously (Metzler-Baddeley et al., 2011, 2012a,b).

Whole brain volume, normalized for head size, was estimated with SIENAX (Smith et al., 2002), part of FSL^2^ (FMRIB Software Library, Version 5.0). White matter lesions were segmented and their total volume quantified using a multispectral image-processing tool, MCMxxxVI (Hernandez et al., 2010).

### Network Construction and Graph Theory-Based Analysis

**FIGURE 1:**
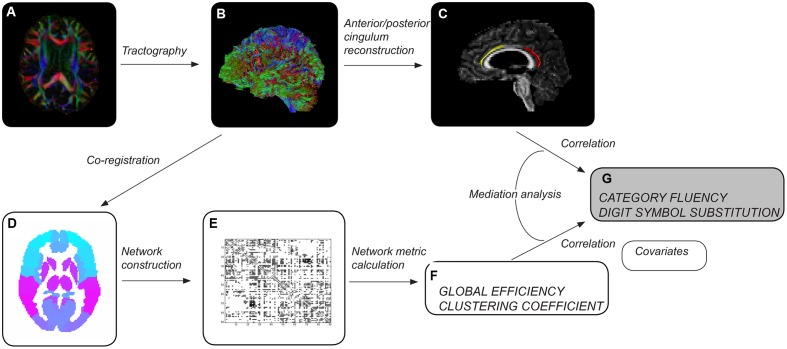
**Overview of methods**. After preprocessing each diffusion tensor imaging dataset **(A)**, whole-brain tractography was performed **(B)**. Cingulum segments of interest were reconstructed **(C)** – left anterior in healthy elderly (yellow), left posterior in patients with mild cognitive impairment (red). Whole-brain tractograms were coregistered to the automated anatomical labeling atlas template **(D)**, consisting of 90 regions corresponding to nodes of the network. The number of reconstructed streamlines between any two regions of the template was used to weight network edges, resulting in a 90 × 90 weighted adjacency matrix **(E)**. Measures of network topology were computed for individual brain networks **(F)**. Tract and network measures were assessed as predictors of cognitive control performance **(G)**. Age, gender, educational attainment, brain volume and volume of white matter hyperintensities were used as covariates.

Whole-brain tract reconstructions were transformed into Montreal Neurological Institute (MNI) space within ExploreDTI, using a non-rigid transformation utilizing B-splines. Gray matter was then parcellated into 90 cortical and subcortical regions, 45 for each hemisphere, using the automated anatomical labeling (AAL) atlas (Tzourio-Mazoyer et al., 2002) (**Figure 1**). Each region was used to define a node of a network graph. Edges were defined by tractography streamlines connecting any pair of nodes. An edge was defined as present between two nodes if a streamline was reconstructed with start and end points in each. Networks were weighted by the number of reconstructed streamlines.

Network metrics were computed using Brain Connectivity Toolbox^3^ (Rubinov and Sporns, 2010). We investigated measures of global and local network architecture: global efficiency, mean clustering coefficient and small-worldness.

### Statistical Analysis

Global efficiency, clustering coefficient and small-worldness were compared between MCI and control groups using unpaired *t*-tests. Associations with cognitive scores were computed in each group separately using Pearson’s product-moment correlation coefficients. Bonferroni correction for multiple comparisons was applied based on the number of network measures. Cognitive measures tend to be strongly correlated with each other and in these circumstances Bonferroni correction is vastly over-conservative, so correction was not applied for the number of cognitive measures. Partial correlation coefficients were calculated accounting for potential confounding variables: age, gender, education (in years), total brain volume, and total white matter lesion volume.

Linear regression models were constructed for Category Fluency and Digit Symbol Substitution task performance to investigate mediation effects. Measures of tract microstructure that were used were based on previously determined associations between Category Fluency and Digit Symbol Substitution, and the microstructure of cingulum segments: left anterior fractional anisotropy in controls, and left posterior mean diffusivity in MCI. These associations were identified in a previous analysis of the same dataset (based on diffusion MRI but not including network graph or graph theory measures), detailed in Metzler-Baddeley et al. (2012a). Separate models were constructed that included: (i) tract microstructure alone; or (ii) both tract microstructure and a single network measure. Thus, the relationships between tract microstructure and cognition, and network topology and cognition were established, and the influence of tract microstructure on cognition while controlling for network topology was assessed. The mediation effect was assessed as a decrease in the value of the standardized regression coefficients (β) for the association between cingulum microstructure and cognition after inclusion of a network measure in the model. Estimates of direct and indirect causal effects were obtained from the models using the non-parametric bootstrapping approach, and the proportion mediated by the network measure was estimated (Imai et al., 2010). This approach allowed measurement of a partial mediation effect and was not aimed at showing full mediation (where inclusion of a mediator leads to a measured association between two factors falling to zero). To test specificity of the investigated relationships for cognitive control, a similar analysis was performed for episodic memory: parallel regression models were constructed with free recall as the dependent variable and fornix tissue volume fraction as the relevant single-tract measure (Metzler-Baddeley et al., 2012b).

Structural equation modeling was performed within the statistical software package R^4^, using an approach analogous to previous studies (Lawrence et al., 2014; Knopman et al., 2015). Tract and network measures were tested for interaction in each model. No significant interaction was found; therefore interaction terms were not included in final models. For terms in all models, variance inflation factors indicated no significant multicollinearity (variance inflation factors <3).

## Results

### Group Comparisons

Demographic, cognitive and general MRI measures for the groups are provided in **Table 1**. Structural networks of both healthy older adults and patients with MCI exhibited small-world topology. There was no difference in small-worldness between groups. In contrast, both global efficiency and mean clustering coefficient were reduced in MCI. On the basis of group differences, global efficiency and mean clustering coefficient were taken forward to analysis of relationships with cognition (leading to Bonferroni-corrected significance equivalent to uncorrected *p* < 0.025).

**Table 1:** Demographic data and group comparison of cognitive scores, MRI measures and measures of network topology.

	Controls	MCI	*t* statistic (df); *p*
Age (yrs.)	74.0 (6.5)	76.8 (7.3)	1.3 (43); 0.19
Education (yrs.)	15 (3)	14 (4)	1.8 (43); 0.08
NART-R IQ	120 (9)	115 (11)	1.8 (43); 0.08
Percentage females	50%	44%	
*Cognitive measures*			
Category fluency	39.5 (10.9)	25.6 (7.9)	**4.9 (41); <0.001**
Verbal fluency	43.2 (13.1)	35.9 (11.3)	1.9 (41); 0.067
Digit symbol substitution	56.5 (18.6)	34.8 (11.9)	**4.6 (40); <0.001**
Stroop suppression	93.4 (19.1)	57.4 (28.2)	**4.7 (40); <0.001**
Tower of London rule violations	1.2 (1.7)	5.8 (4.5)	**4.2 (41); <0.001**
Trails switching	74.0 (31.6)	105.4 (50.7)	**2.4 (42); 0.021**
FCSRT free recall	29.3 (8.4)	12.1 (9.7)	**6.2 (42); <0.001**
CRMT face recognition	23.4 (2.8)	20.0 (3.4)	**3.6 (41); <0.001**
*MRI – general measures*			
NBV (ml)	1,451.4 (57.4)	1,421.7 (57.4)	1.7 (43); 0.091
WML volume (cm)	15.6 (6.7)	19.6 (10.3)	1.5 (41); 0.15
*Structural network properties*			
Global efficiency	0.0260 (0.0021)	0.0239 (0.0036)	**2.6 (39.6); 0.014**
Mean clustering coefficient	18.1 (1.9)	16.7 (2.3)	**2.2 (43); 0.037**
Small-worldness	1.90 (0.26)	1.95 (0.28)	0.63 (43); 0.53

*Data are shown as mean (SD). A cube root transform was applied to white matter lesion volume. Significant differences (*p* < 0.05) are highlighted in bold*.

*MCI – Mild Cognitive Impairment; NART-R – National Adult Reading Test-Revised; FCSRT – Free and Cued Selective Reminding Test; CRMT – Camden Recognition Memory Test; NBV – normalized brain volume; WML – white matter lesion*

### Relationship between Network Metrics and Cognitive Scores

In MCI, both global efficiency and mean clustering coefficient were associated with cognitive control (**Tables 2** and **3**). In contrast, there were no relationships between global network measures and episodic memory performance. Measures of network topology were not correlated with cognitive scores in control participants.

**Table 2:** Univariate relationship between network topology and cognition in patients with MCI and healthy elderly.

	MCI		Controls	
	Eglob	*C*	Eglob	*C*
				*r (p)*
Cognitive control				
Category fluency	**0.56 (0.005)**	**0.61 (0.002)**	0.34 (0.14)	0.20 (0.39)
Verbal fluency	0.17 (0.43)	0.33 (0.12)	0.00 (0.99)	-0.04 (0.87)
Digit symbol substitution	**0.48 (0.022)**	0.40 (0.06)	0.29 (0.23)	0.14 (0.55)
Stroop suppression	0.46 (0.025)	0.21 (0.32)	0.26 (0.29)	0.24 (0.33)
Tower of London rule violations	-0.04 (0.86)	-0.12 (0.57)	-0.04 (0.88)	0.04 (0.88)
Trails switching	-0.17 (0.44)	-0.42 (0.041)	-0.20 (0.40)	-0.28 (0.24)
Memory				
FCSRT free recall	0.28 (0.19)	0.32 (0.13)	0.16 (0.51)	0.0 (0.99)
CRMT face recognition	0.40 (0.05)	0.08 (0.71)	0.16 (0.51)	0.01 (0.96)

*Pearson product-moment correlations (*r*) of cognitive scores with global efficiency (Eglob) and mean clustering coefficient (C), with parenthetical *p*-values. Coefficients shown in bold reach significance after Bonferroni correction for number of network measures (uncorrected *p* < 0.025), but not number of cognitive tests*.

**Table 3:** Relationship between network topology and cognition in patients with MCI and healthy elderly, adjusting for covariates.

	MCI		Controls	
	Eglob	*C*	Eglob	*C*
				*r (p)*
Cognitive control				
Category fluency	0.41 (0.13)	**0.64 (0.011)**	0.23 (0.46)	0.28 (0.35)
Verbal fluency	0.34 (0.22)	0.42 (0.12)	0.02 (0.96)	0.01 (0.96)
Digit symbol substitution	**0.73 (0.002)**	0.49 (0.06)	0.27 (0.38)	0.15 (0.62)
Stroop suppression	**0.64 (0.010)**	0.26 (0.34)	0.37 (0.21)	0.24 (0.42)
Tower of London rule violations	-0.10 (0.74)	-0.23 (0.41)	0.10 (0.74)	0.05 (0.86)
Trails switching	-0.04 (0.89)	-0.53 (0.041)	-0.22 (0.46)	-0.33 (0.28)
Memory				
FCSRT free recall	0.51 (0.05)	0.47 (0.07)	0.03 (0.93)	0.01 (0.96)
CRMT face recognition	0.29 (0.29)	-0.02 (0.95)	0.22 (0.46)	-0.24 (0.42)

*Partial correlation coefficients (*r*) of cognitive scores with global efficiency (Eglob) and mean clustering coefficient (C), covarying for age, gender, education, normalized brain volume, and total volume of white matter hyperintensities, with parenthetical p*-values. Coefficients shown in bold reach significance after Bonferroni correction for number of network measures (uncorrected *p* < 0.025), but not number of cognitive tests.

### Cognitive Control, Global Network Properties, and Individual Tract Structure

In MCI, the inclusion of global network properties led to an attenuation of the relationship between single tract microstructure and cognition (**Tables 4** and **5**). For Category Fluency, both left posterior cingulum microstructure and mean clustering coefficient were significant independent predictors (**Table 5**).

**Table 4:** Regression models for measures of cognitive control in healthy elderly.

	Model 1:	Model 2:		
	Cingulum	Network measure		Cingulum
				*β* *(p)*
Category fluency	0.63 (0.003)	*Eglob*	0.29 (0.11)	0.60 (0.003)
		*C*	0.23 (0.21)	0.64 (0.002)
Digit symbol	0.52 (0.022)	*Eglob*	0.25 (0.23)	0.50 (0.026)
		*C*	0.17 (0.43)	0.53 (0.023)

*Models with fractional anisotropy of the left anterior cingulum (1), and additionally a network measure (2) as predictors. Displayed are standardized regression coefficients (β) with parenthetical *p*-values*.

*Eglob – global efficiency; C – mean clustering coefficient*.

**Table 5:** Regression models for measures of cognitive control in MCI.

	Model 1:	Model 2:		
	Cingulum		Network measure	Cingulum
				*β* *(p)*
Category fluency	-0.66 (0.001)	*Eglob*	0.32 (0.15)	-0.47 (0.037)
		*C*	0.42 (0.020)	-0.49 (0.008)
Digit symbol	-0.52 (0.016)	*Eglob*	0.33 (0.17)	-0.33 (0.18)
		*C*	0.25 (0.25)	-0.42 (0.058)

*Models with mean diffusivity of the left posterior cingulum (1), and additionally a network measure (2) as predictors. Displayed are standardized regression coefficients (β) with parenthetical *p*-values*.

*Eglob – global efficiency; C – mean clustering coefficient*.

**FIGURE 2:**
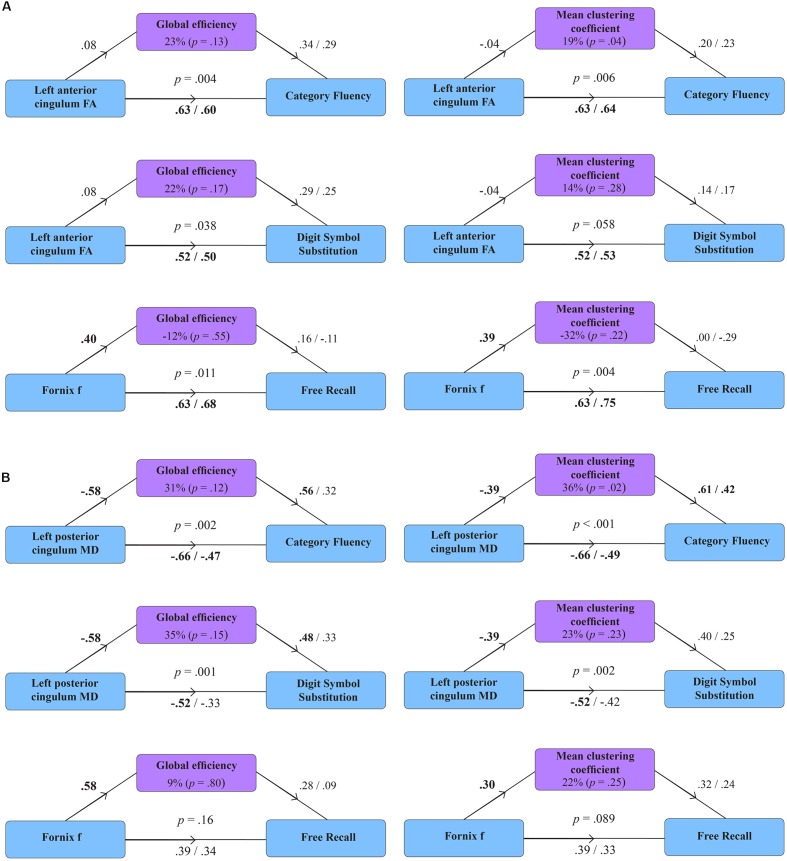
**Mediation models for the effect of global efficiency and mean clustering coefficient in healthy volunteers (A) and patients with MCI (B).** Diagrams present standardized regression coefficients for each path in the model; coefficients after the slash show path values adjusted for the mediation effect. Coefficients in bold correspond to significant associations (*p* < 0.05). *p*-values stand for significance of combined models. The proportion of the effect of tract microstructure (fractional anisotropy – FA; mean diffusivity – MD; tissue volume fraction – f), mediated by the measure of network topology, is displayed as percentage with parenthetical *p*-value, corresponding to the significance of the mediation effect.

**FIGURE 3:**
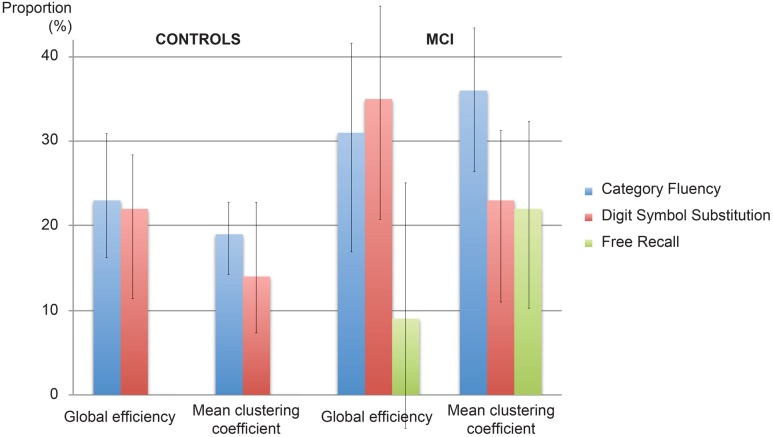
**Proportion of the effect explained by mediation**. The proportion of the effect of cingulum microstructure on cognition mediated by network topology in controls and patients with MCI. Error bars correspond to the interquartile range.

**Figure 2** displays path diagrams of the mediation analysis. The magnitudes of mediation effects are summarized in **Figure 3**. The proportion of the effect of cingulum microstructure on cognitive scores, mediated by global efficiency, varied from 22 to 35% (**Figure 3**). In patients, the mediation effect was strongest for the relationship between left posterior cingulum and Category Fluency, 31% of which was explained by global efficiency (*p* = 0.12) and 36% by mean clustering coefficient (*p* = 0.02). Mean clustering coefficient was also a significant partial mediator of the link between left anterior cingulum and Category Fluency in controls (19% of variance due to mediation effect, *p* = 0.04). Mediation effects of network topology were not demonstrated for episodic memory and the association between fornix structure and free recall, in either of the two groups (**Table 6**; **Figure 3**).

**Table 6:** Regression models for free recall in healthy elderly and MCI.

	Model 1:	Model 2:		
	Fornix	Network measure		Fornix
				*β* *(p)*
Healthy elderly	0.63 (0.003)	*Eglob*	-0.11 (0.58)	0.68 (0.004)
		*C*	-0.29 (0.15)	0.75 (0.001)
MCI	0.39 (0.058)	*Eglob*	0.09 (0.71)	0.34 (0.17)
		*C*	0.24 (0.25)	0.33 (0.11)

*Models with fornix volume fraction (1), and fornix volume fraction and a network measure (2) as predictors. Displayed are standardized regression coefficients (β) with parenthetical *p*-values.*

*Eglob – global efficiency; C – mean clustering coefficient*.

## Discussion

Mild Cognitive Impairment is often considered a prodrome of dementia. We showed previously that microstructure is altered in white matter tracts in MCI and that alterations in specific tracts relate to specific aspects of the cognitive deficit. The present analysis demonstrates that global properties of the structural connectome are also altered. Patients with MCI had reduced global efficiency and mean clustering coefficient, in comparison with matched controls. While whole-brain network measures were not related to episodic memory, measures of network efficiency and clustering were related to cognitive control in MCI. This was the case despite the fact that episodic memory deficits were the most consistent, indeed defining feature of the MCI group. Episodic memory impairment was a prerequisite for the diagnosis while only seven patients with MCI displayed additional executive deficits. This result suggests that global networks are perturbed in MCI, but are not critical to the core deficit in episodic memory, which relates to damage within the relatively narrow and circumscribed extended hippocampal network.

A relationship between network efficiency and executive function has been described in Alzheimer’s disease (Reijmer et al., 2013), but also in other brain disorders such as traumatic brain injury (Caeyenberghs et al., 2012), small-vessel disease (Lawrence et al., 2014), and cerebral amyloid angiopathy (Reijmer et al., 2015). In patients with small-vessel disease and cerebral amyloid angiopathy, network measures were related only to executive function, but not memory performance. However, in these diseases episodic memory deficits are mild or absent, so this dissociation might have been explained by a lack of variance in memory scores. In the present study, conversely, episodic memory was impaired to a greater extent, and more consistently, than cognitive control. This dissociation therefore is more likely to reflect the functional anatomy of cognitive control and episodic memory in the brain and the dependence of cognitive control on a more diffuse network. Further, when correlations were controlled for the volume of white matter lesions, as well as other potential confounders, the pattern of associations remained consistent, and in some cases became stronger, indicating that small vessel disease did not account for the associations observed in this study. Mediation analyses suggested that the relationship between cingulum microstructure and cognitive control was partly mediated by global network topology, while no such link was observed for the relationship between fornix structure and episodic memory. These findings further underline a qualitatively different relationship between tracts and cognitive function for cognitive control and episodic memory.

One intriguing parallel to the pattern of results is that pathological processes also vary in whether they target local structures or more global infrastructure. For example, amyloid and tau pathologies have strong local predilections, at least early in the course of disease. Microvascular disease, on the other hand, leads to diffuse alterations in white matter microstructure so, potentially, it could have a general effect on network efficiency (Lawrence et al., 2014). One strength of the approach taken is that it provides a way to understand how coexistent pathologies could interact. For example, localized neurodegeneration and network-wide effects of diffuse microvascular disease could act synergistically to impair cognitive or executive control.

However, the contrasting relationships of network topology to episodic memory and cognitive control might also be related to methodology used. One possibility is that episodic memory depends on a network that more heavily involves subcortical structures and connections, particularly in the diencephalon, and that in turn topology of these networks is not strongly represented in whole-brain network metrics, constructed using current methods. Parcellation of nodes might be more effective for networks that involve multiple neocortical regions, such as those involved in cognitive control, than for networks with fine-grained subcortical anatomy. The AAL atlas used, as well as alternative parcellation techniques, do not include the mammillary bodies, for example, which are crucial structures within the extended hippocampal network involved in episodic memory.

The pattern of results suggests that damage to a tract such as the cingulum can degrade cognitive performance through two distinct roles of this tract – as a conduit for communication of specific information within a dedicated network for cognitive control, and as a more generic ‘backbone’ for communication across global brain networks. Previous work has shown that hub regions such as the anterior and posterior cingulate cortices, and their connections, might be important not only because they harbor critical functional specializations but also because they mediate connectivity across the structural network more broadly including, for example, in the case of the posterior cingulate cortex, tuning network metastability (Leech and Sharp, 2014).

A limitation of this study, common to studies based on tractography, is the risk of false positive and false negative connections. Weighting of network edges by the total number of reconstructed streamlines should reduce the impact of anatomically spurious edges as, in general, only a few outlier streamlines will run between regions that do not have a true connection. The choice of method for weighting edges is a controversial aspect of the application of graph theory to structural networks. Number of streamlines was used to offer consistency with previous studies and to avoid using microstructural measures known to be abnormal in MCI, but the effect of different weighting approaches has not been investigated in detail. Cognitive control is multifaceted and a number of measures provide overlapping insights into these processes. The Bonferroni method is highly over-conservative in the presence of multiple inter-correlated measures. Correction was therefore applied for number of network measures but not for number of cognitive measures, so that the risk of false positive correlations may not be completely eliminated in the regression analyses. Similarly, a large number of mediation models could have been constructed based on different measures. To minimize the risk of mediation emerging by chance, we selected the two measures most consistently associated with cognition in regression analysis (**Tables 2** and **3**). In addition, a limitation of the mediation analysis performed is that we cannot make definite conclusions on the direction of the effect. Even though it seems less biologically plausible, our results do not exclude the possibility of cingulum microstructure mediating the effect of network topology on cognition.

Further insight into the dynamics of the relationship between ‘local’ and ‘global’ disease-related alterations could be gained by observing our population in a longitudinal setting, or additionally including a group of patients with more severe cognitive impairment. The current study does not extend to brain function, inferred from functional MRI data. It is possible that the topology of structural networks will not be entirely reflected by functional networks, which differ in being dynamic over short time scales. Finally, the interplay between ‘local’ and ‘global’ structural and functional changes might be of interest beyond cognitive function. Functional variation within the cingulate cortex and the large-scale networks might be related to the expression of specific clinical phenotypes, rather than disease-related alterations, such as the occurrence of hyperarousal, anxiety or hallucinations in neurodegenerative disorders (Franciotti et al., 2015). A similar approach could be used to test this hypothesis in Alzheimer’s disease and other neurodegenerative disorders.

Potential treatments such as transcranial magnetic stimulation or direct current stimulation have largely been thought of in terms of localized effects on function. However, a number of studies show that treatment delivered locally can have effects on global network topology and dynamics (Polanía et al., 2011; Shafi et al., 2014). In principle, these wider effects could also be harnessed to restore network function. Our results suggest that for some functions – such as cognitive control – the ideal strategy may involve targeting both local and global alterations in brain structure and function.

## Author Contributions

RB contributed to study conception, data analysis, statistical analysis, writing and editing the manuscript. CM-B contributed to study conception, data collection, writing and editing the manuscript. MAI contributed to data analysis, statistical analysis, writing and editing the manuscript. DKJ contributed to data collection, data analysis, writing and editing the manuscript. MJO contributed to study conception and design, data collection, data analysis, writing, and editing the manuscript.

## Conflict of Interest Statement

The authors declare that the research was conducted in the absence of any commercial or financial relationships that could be construed as a potential conflict of interest.

## Footnotes

1 http://www.exploreDTI.com

2 http://www.fmrib.ox.ac.uk/fsl/

3 https://sites.google.com/site/bctnet

4 http://www.r-project.org
